# Environmental Drivers of Phytoplankton Structure in a Semi-Arid Reservoir

**DOI:** 10.3390/biology14080914

**Published:** 2025-07-22

**Authors:** Fangze Zi, Tianjian Song, Wenxia Cai, Jiaxuan Liu, Yanwu Ma, Xuyuan Lin, Xinhong Zhao, Bolin Hu, Daoquan Ren, Yong Song, Shengao Chen

**Affiliations:** 1 College of Life Sciences and Technology, Tarim Research Center of Rare Fishes, State Key Laboratory Incubation Base for Conservation and Utilization of Bio-Resource in Tarim Basin, Tarim University, Alar 843300, China; 10757213076@stumail.taru.edu.cn (F.Z.); 2485532425zm@gmail.com (W.C.); 10757241109@stumail.taru.edu.cn (J.L.); 119950007@stumail.taru.edu.cn (D.R.); songyongdky@126.com (Y.S.); 2 College of Material Science and Engineering, Beijing University of Chemical Technology, Beijing 100029, China; 3 College of Water Sciences, Beijing Normal University, Beijing 100875, China; 202131470020@mail.bnu.edu.cn; 4 Institute of Fisheries Science, Xinjiang Uygur Autonomous Region, Urumqi 830000, China; myw0012@126.com; 5 Fishery Techincal Extension Station, Xinjiang Production Construction Group, Urumqi 830000, China; btsc229@sina.com (X.L.); btsczxh@126.com (X.Z.); huberlin@126.com (B.H.)

**Keywords:** arid zone hydroecology, community spatial heterogeneity, identification of key taxa, differences in ecological response

## Abstract

Artificial reservoirs in arid regions provide unique ecological environments for studying the spatial and functional dynamics of plankton communities under the combined stressors of climate change and anthropogenic activities. This study conducted a systematic investigation of the phytoplankton community structure and its environmental drivers in 17 artificial reservoirs in the Ili region of Xinjiang in August and October 2024. The Ili region is located in the temperate continental arid zone of northwestern China. A total of 209 phytoplankton species were identified, with Bacillariophyta, Chlorophyta, and Cyanobacteria comprising over 92% of the community, indicating an oligarchic dominance pattern. The decoupling between numerical dominance (diatoms) and biomass dominance (cyanobacteria) revealed functional differentiation and ecological complementarity among major taxa. Through multivariate analyses, including Mantel tests, principal component analysis (PCA), and redundancy analysis (RDA), we found that phytoplankton community structures at different ecological levels responded distinctly to environmental gradients. Oxidation-reduction potential (ORP), dissolved oxygen (DO), and mineralization parameters (EC, TDS) were key drivers of morphological operational taxonomic unit (MOTU). In contrast, dominant species (SP) were more responsive to salinity and pH. A seasonal analysis demonstrated significant shifts in correlation structures between summer and autumn, reflecting the regulatory influence of the climate on redox conditions and nutrient solubility. Machine learning using the random forest model effectively identified core taxa (e.g., MOTU1 and SP1) with strong discriminatory power, confirming their potential as bioindicators for water quality assessments and the early warning of ecological shifts. These core taxa exhibited wide spatial distribution and stable dominance, while localized dominant species showed high sensitivity to site-specific environmental conditions. Our findings underscore the need to integrate taxonomic resolution with functional and spatial analyses to reveal ecological response mechanisms in arid-zone reservoirs. This study provides a scientific foundation for environmental monitoring, water resource management, and resilience assessments in climate-sensitive freshwater ecosystems.

## Simple Summary

Understanding how plankton communities respond to environmental changes is essential for managing freshwater ecosystems, especially in arid regions. In this study, we investigated phytoplankton’s composition, distribution, and environmental responses in 13 reservoirs in Ili, Xinjiang, a typical arid region in northwest China. We identified 209 species, with diatoms, green algae, and cyanobacteria dominating the communities. Seasonal variation and spatial heterogeneity were observed in species composition and environmental factors such as temperature, redox potential, and salinity. Using multivariate statistics and machine learning, we revealed that key taxa dominate in shaping community structures and can serve as water quality indicators. Our results demonstrate that phytoplankton communities in arid-zone reservoirs exhibit distinct spatial patterns and respond differently to environmental gradients at taxonomic and functional levels. This study provides a scientific basis for biodiversity assessment and ecological management in dryland freshwater ecosystems.

## 1. Introduction

Reservoirs are globally widespread artificial water bodies that serve essential ecological and economic functions, including water supply, irrigation, aquaculture, flood control, and power generation [1]. According to the Global Reservoir and Dam Database (GRanD), over 6800 reservoirs collectively store more than 6000 km^3^ of water. Unlike natural lakes and rivers, reservoirs exhibit unique hydrological and ecological characteristics, which shape distinct patterns in plankton community structure and function [2]. As key components of aquatic ecosystems, phytoplankton and zooplankton are fundamental to primary and secondary productivity, influencing material cycling, energy flow, and the overall stability of the food web [3].

Phytoplankton, as primary producers, convert solar energy into organic matter through photosynthesis, supporting higher trophic levels and regulating oxygen and carbon dynamics [4]. Zooplankton, as secondary producers, link phytoplankton to larger consumers while contributing to water clarity and ecosystem health by grazing on algae, including harmful cyanobacteria [5]. Both groups are highly sensitive to environmental changes, and their dynamics serve as indicators of trophic status and ecological integrity in freshwater systems. Traditional studies of plankton communities have focused on taxonomic compositions and environmental correlations. However, the ecosystem functions of plankton are shaped not only by diversity but also by interspecific interactions such as predation, competition, and mutualism. To address this complexity, Ecological Network Analysis (ENA) has emerged as a powerful approach for quantifying species interactions, identifying keystone taxa, and assessing ecosystem stability [6,7]. ENA enables the construction of species co-occurrence networks and functional webs, providing insights into ecosystem robustness and responses to disturbances, such as climate change and anthropogenic stress.

Recent research using ENA has significantly advanced our understanding of plankton dynamics in large lakes, eutrophic systems, and regions impacted by climate change. For instance, studies in the North American Great Lakes demonstrated how invasive species reshaped trophic interactions and reduced network stability [8]. European and Asian studies showed how warming and nutrient enrichment alter energy pathways through shifts in phytoplankton–zooplankton linkages [9,10,11]. However, most of these efforts have concentrated on temperate, humid zones, with limited attention to arid environments. The Ili region in Xinjiang presents a unique setting for such investigations due to its complex topography, semi-arid climate, and strong anthropogenic influences, including reservoir regulation, irrigation, and aquaculture [12]. These factors jointly shape the plankton community structure and ecosystem functioning. Therefore, exploring the ecological networks of plankton in this region is crucial for understanding biodiversity patterns and guiding sustainable water resource management in arid-zone freshwater systems.

The main significance of this study includes the following aspects: (1) To resolve the interaction patterns among populations and identify key species and their ecological functions. (2) To analyze the robustness of the plankton network in the reservoir and to explore the driving effects of water quality factors, climate change, and human activities on the network structure. (3) To optimize ecological restoration and water quality management strategies in reservoirs to promote the sustainable use of water resources. (4) To fill the gap of aquatic ecological network research in the arid zone and to provide scientific references for the management of water bodies in similar ecological environments worldwide.

## 2. Materials and Methods

### 2.1. Study Area

The Ili region of Xinjiang, situated on the northern slopes of the Tianshan Mountains, features a temperate continental climate moderated by orographic effects and westerly winds, resulting in relatively high humidity and precipitation compared to other arid areas in Xinjiang. Annual rainfall ranges from 200 to 800 mm, concentrated in spring and summer, while the yearly temperature varies between 4 °C and 10 °C, with extremes ranging from below −30 °C in winter to above 35 °C in summer [13]. These pronounced seasonal fluctuations shape reservoir hydrology and influence plankton dynamics.

The region has a well-developed fluvial system, with major rivers such as the Ili, Tekes, and Gongnaisi feeding numerous reservoirs, which are primarily located in the Ili River Valley and adjacent terrains. Most reservoirs are transformed from natural water bodies and serve multiple purposes, including irrigation, aquaculture, and water regulation. Due to seasonal precipitation variability, reservoirs heavily rely on snowmelt and upstream inflows for replenishment, functioning as vital flood control systems during wet periods and as supply sources during dry seasons.

The interplay of topography, climate, and human activities shapes reservoir environments in the region. Varied landforms—from alpine valleys to lowland plains—result in heterogeneous water conditions, affecting nutrient levels, light penetration, and biological productivity. High-altitude reservoirs typically have clearer water and lower nutrient input, while lowland systems are more susceptible to eutrophication due to agricultural runoff. Surrounding land use, including grasslands, forests, and croplands, further modulates water quality and plankton community composition [14].

Human influences are particularly significant. Agricultural water withdrawal causes fluctuations in reservoir levels, destabilizing aquatic habitats. Additionally, intensive aquaculture increases organic loads, altering nutrient dynamics and phytoplankton–zooplankton interactions. Overall, the plankton communities in Ili reservoirs are co-regulated by hydrological variation, climatic conditions, and anthropogenic pressures, making the region an ideal model for studying ecological responses in arid-zone artificial water bodies.

### 2.2. Field Sampling and Data Acquisition

According to the Technical Methods for Ecological Investigation of Rivers, the spatial distribution of sampling points should be sufficiently representative to quantitatively reflect all the main features of water quality, sediment, and ecosystems. Generally, this involves deployment in areas with less artificial landscape, 200 m upstream and downstream of the river confluence and bridge piers and other artificial landscapes, with full consideration given to the diversity of habitats in the sample points, with different habitats such as shallow water on both sides of the river bank, deep water in the middle of the river, aquatic grassy areas, and spacious water surface areas.

**Figure 1:**
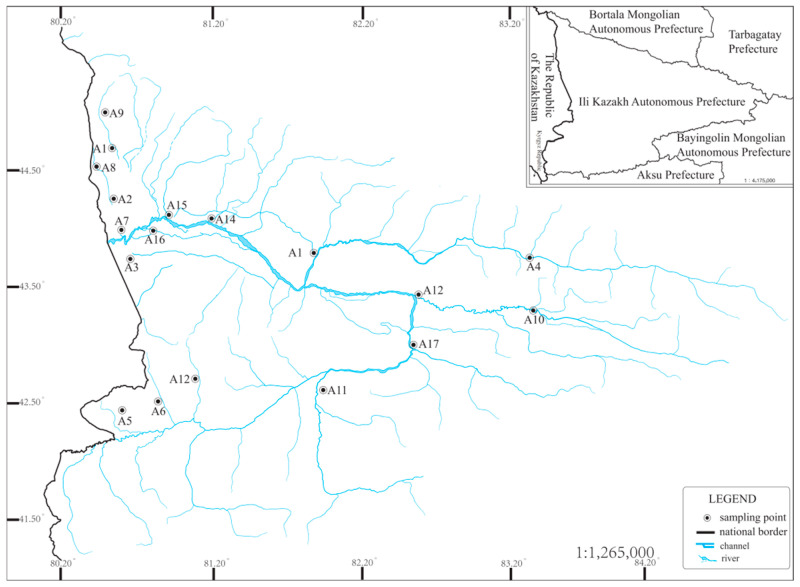
Sampling point location diagram, A1–A17 in the figure are sampling sites.

According to the references and on-site surveys, combined with this study’s survey requirements, the selection of survey sections can meet the requirements of collecting as many aquatic organisms as possible and the survey requirements. The most basic conditions for collecting as many aquatic organism samples as possible, as well as the accessibility of vehicles and people, must be met. The sampling section should also meet the accessibility of vehicles and people, i.e., the collectability. Based on the above conditions, 17 sampling transects were set up (Figure 1), and the sampling time was August and October 2024 to collect aquatic organism samples and water quality samples in two seasons, respectively. In order to ensure the accuracy and timeliness of the survey, a handheld GPS (GPSMAP 67, Garmin Corp., Beijing, China) was used to locate the surveyed transects for each survey, and in situ monitoring of water quality in the transects was also carried out.

At each point, water quality samples and phytoplankton samples were collected from the surface layer (0.5 m below the water surface), the middle layer (15 to 21 m below the water surface), and the bottom layer (30 to 35 m below the water surface). At each sampling point, 1 L of water sample was taken with a water collector (CS-100S, Ruibin Technology Co., Ltd., Guangzhou, China), fixed with 10 mL of 5% Ruger’s solution (DM0022, Leagene, Biotechnology Co., Ltd., Beijing, China). The sedimentation was left to settle for 24 h, concentrated to 30~50 mL, and then sent back to the laboratory to take 0.1 mL in a phytoplankton counting frame for species identification and cell counting under a microscope (OlympusCX21, Mshot Photoelectric Technology Co., Ltd., Guangzhou, China) at 400×. The phytoplankton species identification and quantification (density and biomass calculation) were based on relevant literature, and all individuals were identified to the lowest taxonomic unit possible [15,16,17]. Phytoplankton biomass is measured in wet weight g/L, and density is measured in cells/L. The classification of phytoplankton followed standard Chinese taxonomic systems. However, it is acknowledged that in modern phylogenetic frameworks, *Bacillariophyta* and *Chrysophyta* are now considered part of *Heterokontophyta* (*Stramenopiles*). For clarity and consistency with national references, we retained these terms in the main text, with taxonomic updates discussed where relevant. Water temperature (WT, °C), electrical conductivity (EC, μs/cm), salinity (Sal, ppt), pH, dissolved oxygen (DO, mg/L), total dissolved solids (TDS, mg/L), and oxidation-reduction potential (ORP, mV) were measured in situ at each site using a multiparameter water quality detector (YSI 556MPS, YSI Group, Beijing, China).

### 2.3. Data Analysis

To assess the relationships between phytoplankton community composition and environmental factors, Redundancy Analysis (RDA) was conducted using the vegan package in R (4.4.3) Before RDA, Detrended Correspondence Analysis (DCA) was applied to determine gradient lengths and select the appropriate ordination method. Since the longest DCA axis was <3.0, a linear method (RDA) was deemed suitable.

RDA was used to visualize the ecological response of phytoplankton groups (MOTUs) to physicochemical parameters (e.g., ORP, pH, EC, and Sal). The analysis was performed on Hellinger-transformed abundance data. The statistical Significance of the RDA model and individual environmental variables was tested using Monte Carlo permutation tests (999 permutations). Environmental variables with *p* < 0.05 were considered significant drivers.

To further evaluate the correlation between phytoplankton community structure and environmental matrices, Mantel tests were performed using Pearson’s correlation with 999 permutations, controlling for spatial autocorrelation through a geographic distance matrix.

A Random Forest Model (RFM) was applied to identify key phytoplankton taxa responsible for site differentiation. The importance of each taxon was assessed using Mean Decrease Accuracy (MDA), and results were visualized through abundance heatmaps and variable importance plots. Taxa with the highest MDA scores were designated as indicator species.

Dominance degrees of individual phytoplankton species were calculated using the following formula:

Y = (ni/N) × fi,

where ni is the total abundance of species i, N is the total abundance of all species, and fi is the frequency of occurrence of species i across sites. Species with dominance index Y > 0.02 were defined as dominant species (SP1–SP5). Broader morphological groups were aggregated into MOTUs based on taxonomic phylum and shared morphological traits.

## 3. Results

### 3.1. Taxonomic Composition and Structural Features of Phytoplankton Communities

**Figure 2:**
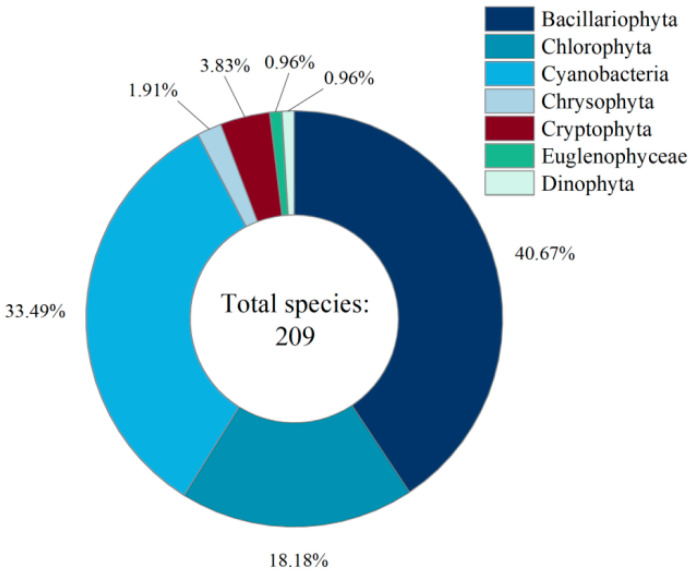
Species composition and proportion of phytoplankton phyla distribution in reservoirs of the Ili region, Xinjiang.

The taxonomic composition and species distribution of phytoplankton in the study area are presented in Figure 2. A total of 209 phytoplankton species were identified, belonging to seven phyla, with Bacillariophyta as the dominant group, accounting for a total of 40.67% of the total, and occupying a dominant position in the composition of the species, followed by Chlorophyta and Cyanobacteria, which accounted for 33.49% and 18.18%, respectively, and constituted the main structure of the community. The total proportion of the three groups reached 92.34%, showing obvious ‘oligarchic’ community characteristics.

The proportion of other groups was relatively low, with *Cryptophyta* accounting for 3.83%, *Chrysophyta* accounting for 1.91%, and *Euglenophyceae* and *Dinophyta* accounting for the least, both with a proportion of 0.96%. This indicates that the structure of the phytoplankton community in this region is centered on a few dominant taxa, with strong stability in species composition. The distribution of species of non-dominant taxa is more sporadic, which may be limited by the differences in environmental conditions or ecological niche adaptability.

Table 1 shows the five dominant phytoplankton species and their dominance degrees in the study area, among which *Limnothrix redekei* (Van Goor) Meffert (SP5) has the highest degree of dominance of 0.24, which is dominant; *Nitzschia palea* (Kützing) W. Smith (SP1) has a degree of dominance of 0.07, which is representative to a certain extent; the remaining three species (SP2–SP4) have a degree of dominance of 0.02, which shows a weak community dominance. Nitzschia palea (SP1) had a degree of dominance of 0.07, which was somewhat representative; the remaining three species (SP2–SP4) had a degree of dominance of 0.02, which showed weak community dominance, and the overall community structure was characterized by ‘dominance by a few dominant species’.

**Figure 3:**
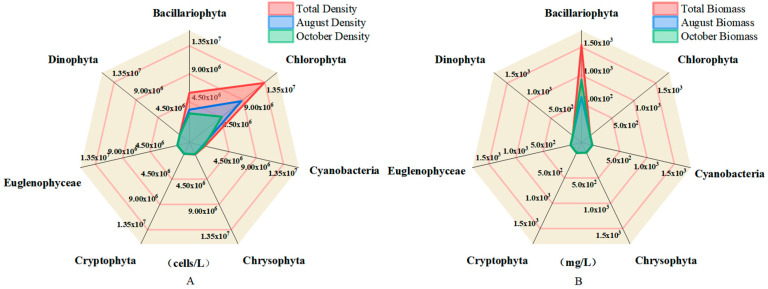
Radar plots of density and biomass distributions of different phytoplankton species; (**A**) indicates the density distribution, and (**B**) indicates the biomass distribution.

To further illustrate the structural differences within the phytoplankton community, radar plots were used to visualize the taxonomic composition in terms of both density and biomass across seasons (Figure 3A,B). In terms of cell density (Figure 3A), Bacillariophyta overwhelmingly dominated the assemblages during both August and October, with cell abundances far surpassing those of other groups, indicating their consistent numerical advantage under varying environmental conditions.

In contrast, biomass-based composition (Figure 3B) revealed a different pattern. Although Bacillariophyta maintained a high density, its contribution to total biomass was relatively low due to a smaller cell size. *Cryptophyta*, particularly in October, exhibited disproportionately high biomass, suggesting a functional shift likely linked to larger cell volume and higher per-cell carbon content. This divergence between density and biomass patterns points to a decoupling between quantitative and qualitative dominance within the phytoplankton community.

**Figure 4:**
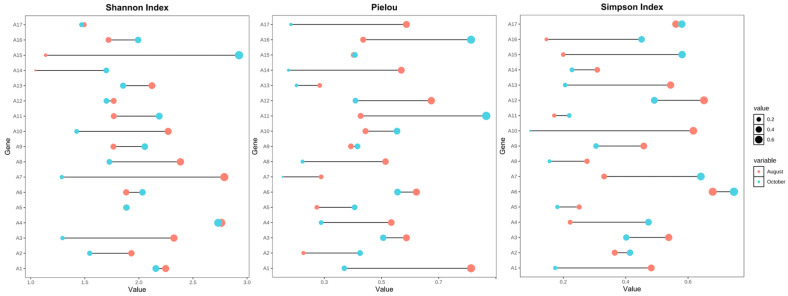
Seasonal variation in phytoplankton α-diversity across 17 sampling sites in the Ili Reservoir. Dot plots show changes in the Shannon index (**left**), Pielou evenness (**middle**), and Simpson index (**right**) between August (red) and October (blue). Circle size corresponds to index values.

Figure 4 illustrates the seasonal variation in α-diversity indices (Shannon index, Pielou evenness, and Simpson index) across 17 sampling sites during August and October. Overall, the Shannon index (left panel) showed a moderate decline from August to October at most sites, indicating a reduction in phytoplankton species richness and evenness as autumn progressed. This trend was particularly evident at sites A13–A17.

Pielou’s evenness index (middle panel) also displayed a general decreasing pattern in October, suggesting that phytoplankton communities became more unevenly distributed, potentially reflecting the dominance of a few taxa during late-season succession. However, some sites (e.g., A6, A8, and A10) exhibited minor or no seasonal change, implying localized environmental buffering effects.

The Simpson index (right panel), which emphasizes dominance patterns, varied across sites but generally decreased in October, indicating increased dominance by specific taxa and reduced community diversity. Notably, sites A2, A3, and A14 showed marked reductions in Simpson diversity, aligning with changes observed in community structure and biomass distribution.

### 3.2. Characterization of the Environmental Response and Spatial Structure of Phytoplankton Communities

Seasonal variation in water environmental parameters was evident across the sampling periods (Table 2). Water temperature (WT) showed a significant decline from August (21.82 ± 2.40 °C) to October (14.62 ± 1.90 °C), with a highly significant difference (F = 115.72, *p* < 0.001). Dissolved oxygen (DO) increased markedly from 6.42 ± 1.14 mg/L to 7.86 ± 0.92 mg/L (F = 28.73, *p* < 0.001), while oxidation-reduction potential (ORP) also rose significantly (F = 5.94, *p* = 0.024). The pH value was significantly lower in October than in August (F = 14.83, *p* < 0.01), indicating increased acidity. In contrast, conductivity (EC), salinity (Sal), and total dissolved solids (TDS) exhibited minor seasonal fluctuations with no significant differences (*p* > 0.05), suggesting relatively stable ionic conditions in the reservoir.

**Figure 5:**
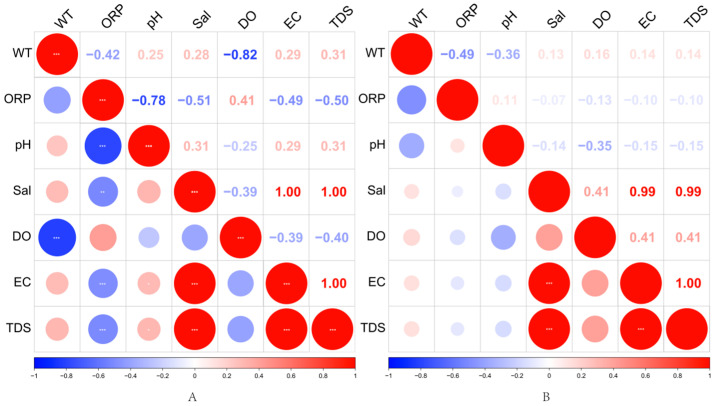
Heat map of correlations between environmental factors in water bodies in August (**A**) and October (**B**); color shade and circle size represent the strength of correlation coefficients, with blue indicating negative correlation and red indicating positive correlation, and significance levels marked by ‘***’ (*p* < 0.001), ‘** ‘marking (*p* < 0.01), and ‘*’ marking (*p* < 0.05).

The heat map analysis of environmental factors in water bodies in August (A) and October (B) in Figure 5 showed some differences in the correlations of environmental factors in water bodies in different seasons. Firstly, water temperature (WT) and oxidation-reduction potential (ORP) were negatively correlated in both periods. However, the correlation was weaker in August (−0.42) and more significant in October (−0.49), suggesting that the decrease in temperature enhanced the redox effect in the water body. In addition, ORP showed a strong negative correlation with pH in August (−0.78), while it was only −0.36 in October, suggesting that the redox state of the water body had a weakened effect on pH in autumn.

Dissolved oxygen (DO) showed the strongest negative correlation with water temperature in August (−0.82), while there was almost no significant correlation in October (0.16), suggesting that DO was significantly regulated by water temperature during the high-temperature period, while other factors more influenced changes in DO during the low-temperature period. Salinity (Sal) remained highly positively correlated with electrical conductivity (EC) and total dissolved solids (TDS) in both periods (both 1.00 or close to 1.00), suggesting that the trends of these parameters were consistent.

In addition, EC and TDS maintained very high positive correlations in August (1.00) and October (0.99), indicating that the trends of these two indicators were highly consistent across seasons. However, the negative correlation of DO with EC and TDS was more pronounced in August (around −0.40), while the correlation was weaker in October (−0.10 to −0.15), suggesting that seasonal variations may have weakened the effect of mineralization on DO. Overall, the effect of water temperature on DO and ORP was more significant in August, while the effect of ORP on water body physicochemical factors weakened in October, but the correlation between EC and TDS remained stable. These results revealed the seasonal variation characteristics of environmental factors in the water body of Yili Reservoir, Xinjiang, which is important for understanding the ecological processes of water bodies in arid regions.

**Figure 6:**
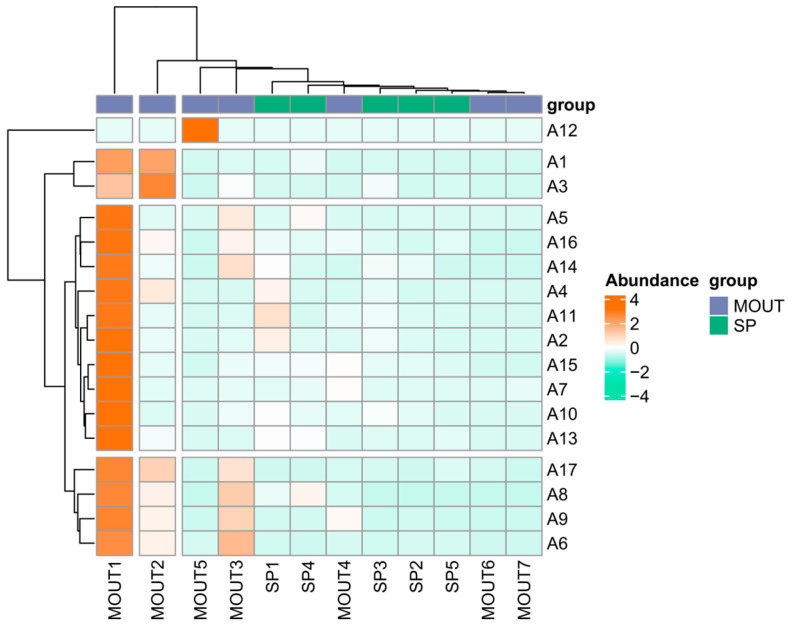
Heatmap showing the correlation pattern between sampling sites (A1–A17) and phytoplankton groups, including morphological taxonomic units (MOTUs) and dominant species (SPs), based on their distribution profiles. Color gradients from green (negative correlation) to red (positive correlation) indicate the strength and direction of correlations. The horizontal axis represents phytoplankton groups, while the vertical axis indicates sampling sites. The dendrograms reflect hierarchical clustering based on community composition similarity.

From the abundance distribution in Figure 6, MOTU1 showed an obvious high abundance in most sampling sites, especially in sites such as A12, A1, and A3, with values close to the highest abundance class (4), indicating its broad dominance in the overall phytoplankton community. MOTU2 and MOTU5 also showed moderate abundance (with values of about 2) in a few sites, but with less extensive spatial distribution than MOTU1. MOTU3, on the other hand, although showing moderate abundance at some sites (e.g., A12), had low or near-zero abundance in most other sample sites.

In contrast, MOTU4, MOTU6, and MOTU7 were generally low in abundance in all sample sites and did not show significant geographic or ecological dominance. Among the dominant species (SP), the overall abundance level was generally low and relatively concentrated in the spatial distribution, while SP1 and SP4 were slightly higher in individual sample sites (e.g., A5, A16), but did not reach the dominance of the main MOTU taxa.

The clustering results showed that the MOTU taxa were clustered into one group, while the SP taxa were clustered into another group, and the divergence between them was obvious. This clustering structure indicates that there are obvious differences in abundance performance and spatial response between phytoplankton community structure and dominant species, and a few phyla in the MOTU taxa dominate multiple sites, whereas the dominant species are more localised and sparse, and do not form a uniform dominant pattern.

The results show that the phytoplankton community has the ecological characteristic of ‘wide distribution of core taxa and localisation of dominant species’, i.e., a few MOTU taxa dominate at multiple sites, while the spatial distribution and abundance of dominant species are relatively limited. This structural difference may be driven by a combination of environmental conditions in the water column, trophic status, and differences in species’ ecological niches.

**Figure 7:**
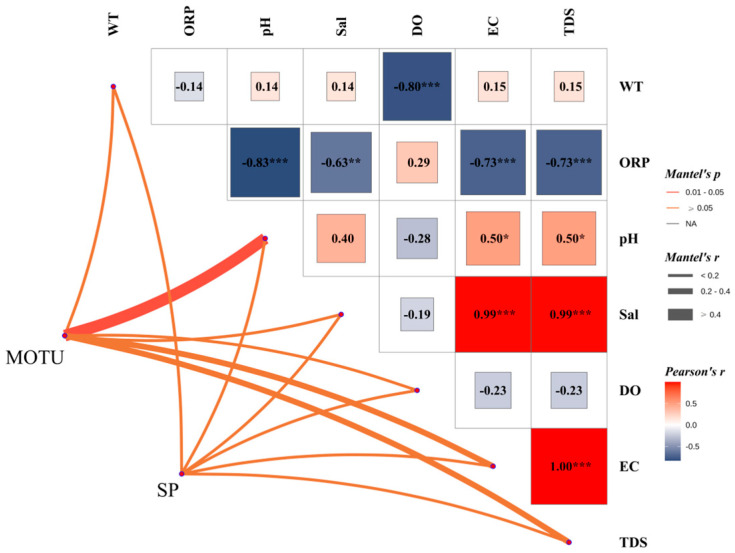
Mantel correlation analysis plot of phytoplankton community structure (MOTU) and dominant species (SP) against environmental factors; the plot shows the correlation between phytoplankton phyla (MOTU) and dominant species (SP) and environmental factors in the water column. The upper panel shows the Pearson correlation coefficient matrix, with the colors indicating the strength of the correlation (red for positive correlation and blue for negative correlation), and significance is indicated by *: indicates a *p*-value less than 0.05; **: indicates a *p*-value less than 0.01; ***: indicates a *p*-value less than 0.001.

Figure 7 At the overall community level (MOTU), phytoplankton structure was significantly negatively correlated with oxidation-reduction potential (ORP) (Mantel’s r = −0.83, *p* < 0.001). It significantly negatively correlated with dissolved oxygen (DO) (r = −0.80, *p* < 0.001) and electrical conductivity (EC) (r = −0.73, *p* < 0.001), and total dissolved solids (TDS) (r = −0.73, *p* < 0.001) were also significantly negatively correlated, suggesting that the redox state and mineralization of the water column strongly regulated phytoplankton community structure.

At the level of dominant species (SP), its structure was mainly positively affected by salinity (Sal) significantly (r = 0.99, *p* < 0.001). Also, it showed a highly significant positive correlation with EC and TDS (r = 0.99, *p* < 0.001), suggesting that the distribution of the dominant species was closely related to the ionic concentration of the water column. In addition, some degree of positive correlation was observed between pH and SP (r = 0.50, *p* < 0.05), suggesting that alkaline environments may favor dominant species.

Notably, there was no significant correlation between MOTU and pH, and no significant relationship was shown between SP and DO (r = −0.23), reflecting that the phytoplankton community was more sensitive to dissolved oxygen. At the same time, the dominant species were more driven by salinity and mineralization factors. Overall, phytoplankton community structure was negatively correlated with oxidative and mineralization indices. At the same time, the distribution of dominant species was significantly positively correlated with salinity and related indices, showing the variability in the response of different ecological strata to environmental factors.

### 3.3. Multivariate Analysis of Phytoplankton Environmental Response

**Figure 8:**
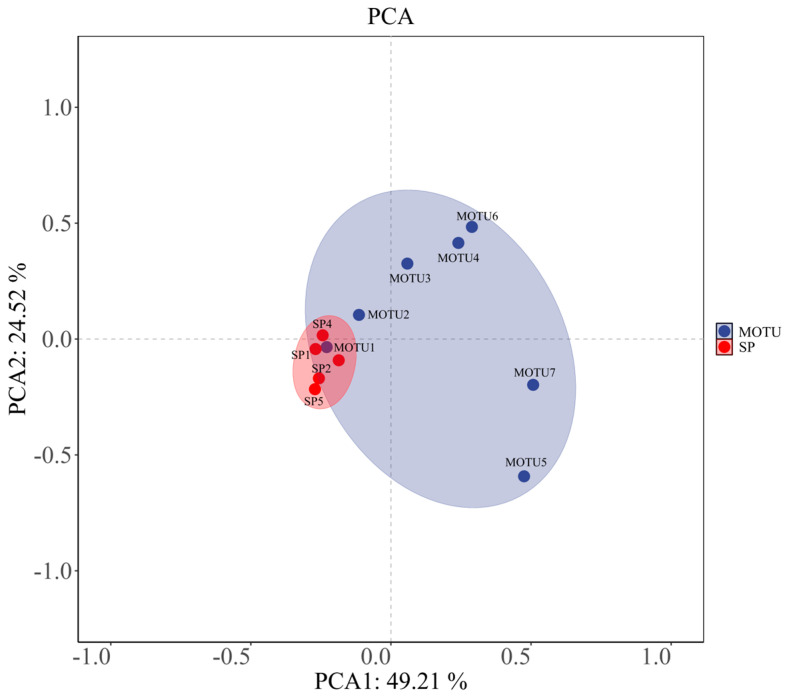
Principal component analysis (PCA) distribution of phytoplankton community (MOTU) and dominant species (SP); the figure shows the distribution characteristics of phytoplankton phyla (MOTU, blue) and dominant species (SP, red) on the first two axes of PCA. Color ellipses indicate 95% confidence intervals.

The distributional characteristics of the phytoplankton community (MOTU) and the dominant species (SP) under principal component analysis (PCA) to assess the differences in their responses driven by the environmental factors are presented in Figure 8. The first two axes of PCA explained a total of 73.73% of the total variance, with principal component 1 (PC1) explaining 49.21%, and principal component 2 (PC2) explaining 24.52%, indicating that the model can effectively summarize the variation between samples.

From the distribution map, MOTU (blue) and SP (red) form separated clustering zones in the two-dimensional space, and the distribution of their respective points is relatively concentrated, constituting two elliptical trust zones, respectively. This spatial segregation indicates that the overall community structure of phytoplankton (MOTU) and dominant species (SP) composition show obvious differences in response patterns under environmental factors. Among them, SP points were highly clustered, indicating that the dominant species had more consistent response characteristics to major environmental gradients. At the same time, MOTU samples were dispersed along the PC1 direction, reflecting the existence of richer ecological differentiation among different phyla under the action of environmental factors.

The PCA results verified differences in the magnitude and direction of the response of different ecological strata (community vs. dominant species) of the phytoplankton community across environmental gradients, further supporting the selective role of environmental factors revealed in the aforementioned Mantel analysis. Such structural differences provide supportive evidence for understanding the differentiation of phytoplankton ecological functions and the environmental driving mechanisms in the water column.

**Figure 9:**
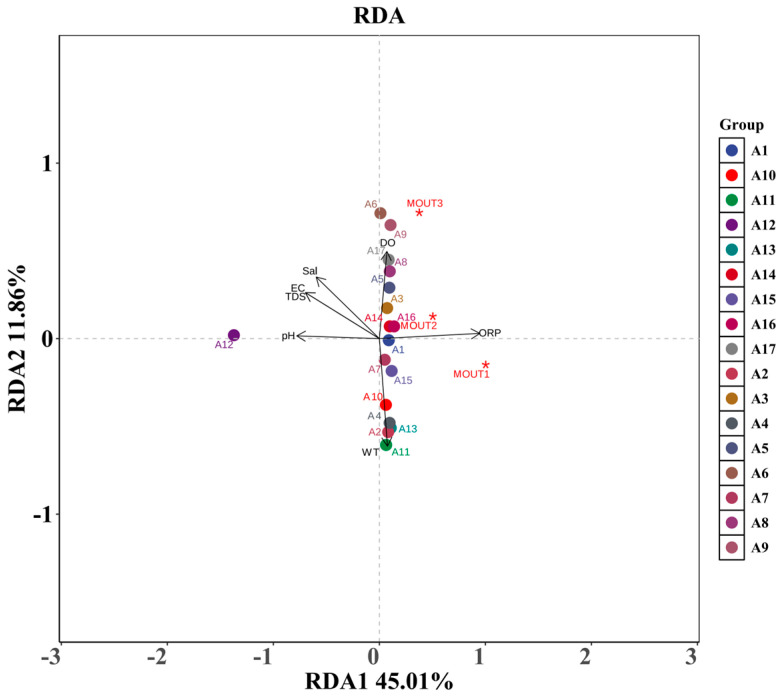
Redundancy analysis (RDA) ordination of phytoplankton phyla (MOTUs) with environmental factors; the figure shows the RDA ordination results of phytoplankton phyla (marked by red *) and sampling sites (A1–A17) with a gradient of environmental factors. Arrows indicate the direction and intensity of the effect of environmental factors on community structure.

The results of redundancy analysis (RDA) between phytoplankton communities (MOTUs) and environmental factors for revealing the response relationships of each phytoplankton phylum under different environmental gradients are presented in Figure 9. The RDA1 and RDA2 axes explained 45.01% and 11.86% of the variance, respectively, and in total, explained 56.87% of the environmental relationships of the communities, suggesting that the major environmental factors have strong explanatory power.

From the figure, it can be seen that the phytoplankton phyla (marked by red *) had obvious differences in spatial distribution. Among them, MOTU1 was consistent with the direction of oxidation-reduction potential (ORP), indicating that ORP strongly drove its distribution; MOTU2 and MOTU3 were located in the transition zone between ORP and other factors, which might be affected by the combined influence of multiple factors at the same time. In contrast, the distribution of other MOTU points was close to the center of the coordinates, indicating that their responses to environmental gradients were weaker or more balanced.

The length and direction of the arrows of each environmental factor reflect the strength and direction of its influence on community structure. **ORP, pH, and water temperature (WT)** arrows are longer, indicating that they are the main driving factors, among which the direction of ORP influence is consistent with multiple sampling sites (e.g., A1, A3, A16, etc.). **Salinity (Sal), dissolved oxygen (DO), electrical conductivity (EC) and total dissolved solids (TDS)**, on the other hand, were concentrated on the left side of the graph, which appeared to be another environmental gradient axis, mainly affecting the sites located in the lower-left corner of the graph, e.g., A12, which was far away from the main clustering area, indicating that the environmental conditions of its water differed significantly from the other sample sites.

Different phytoplankton phyla (MOTUs) showed divergent responses to environmental factors. ORP is one of the most dominant drivers, and marginal sample sites such as A12 may represent special water environmental conditions or extreme ecological niches. The RDA results revealed that the phytoplankton community structure was influenced by the combined effects of multiple physicochemical factors in the water column, reflecting a community pattern shaped by ecological niche differentiation and environmental selection.

### 3.4. Identification of Key Phytoplankton Taxa and Ecological Response Analysis Based on a Random Forest Model

**Figure 10:**
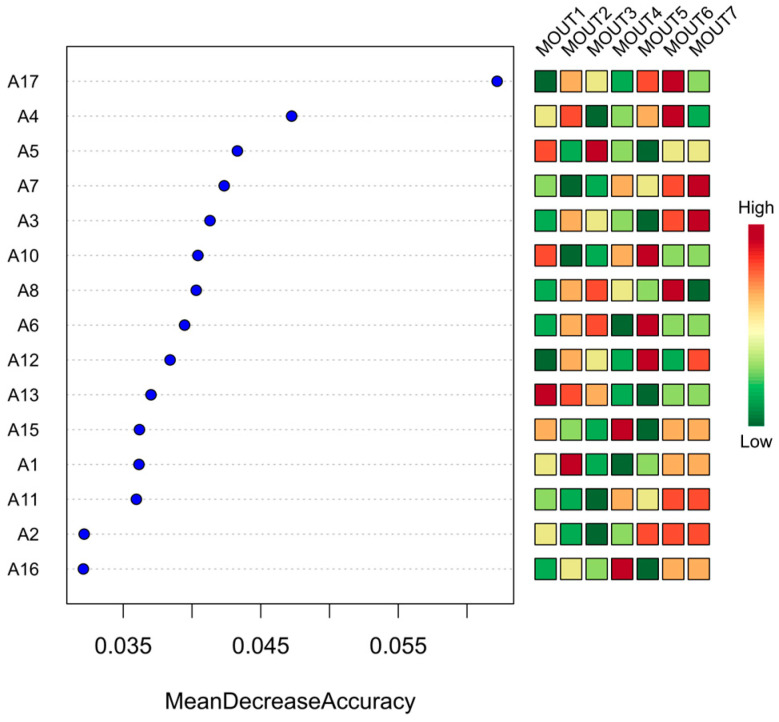
Discriminant importance and abundance distribution of phytoplankton phyla (MOTUs) based on the Random Forest Model (RFM); on the left side of the figure is the ranked discriminant importance of phytoplankton phyla (MOTUs) in the random forest model (as measured by the average decrease in the accuracy), and on the right side of the figure is the standardized abundance heatmap of each MOTU among the 17 sampling points (A1–A17), with the colors indicating the abundance gradient from green (low) to red (high), and the color from green (low) to red (high). (low) to red (high), indicating the abundance gradient.

From the model results presented in Figure 10, MOTU1 has the highest importance, with a much higher average accuracy drop than the other MOTUs, suggesting that it plays the most crucial role in the classification. Secondly, MOTU2, MOTU3, MOTU4, and MOTU5 also have high variable importance and are all in the top five, and these gates are the core variables for the model to distinguish between different homogeneous sites. In contrast, the lower-ranked ones, such as MOTU4 and MOTU5, contribute less to the model accuracy improvement, and the average predictive contribution value is close to zero, indicating that they are less indicative.

In the abundance heatmap, MOTU1 exhibits high abundance (red) in multiple sample points (e.g., A4, A5, A13, A15, etc.) and low abundance (green) in some sample points (e.g., A12 and A17), showing obvious spatial differentiation. Both MOTU2 and MOTU3 demonstrated site-specific abundance patterns, being dominant in some areas while nearly absent in others. In contrast, MOTU14 and MOTU15 showed relatively low abundance in all sample sites, and the color patches were mostly green or light yellow with little variation, further confirming their lower ecological response sensitivity.

**Figure 11:**
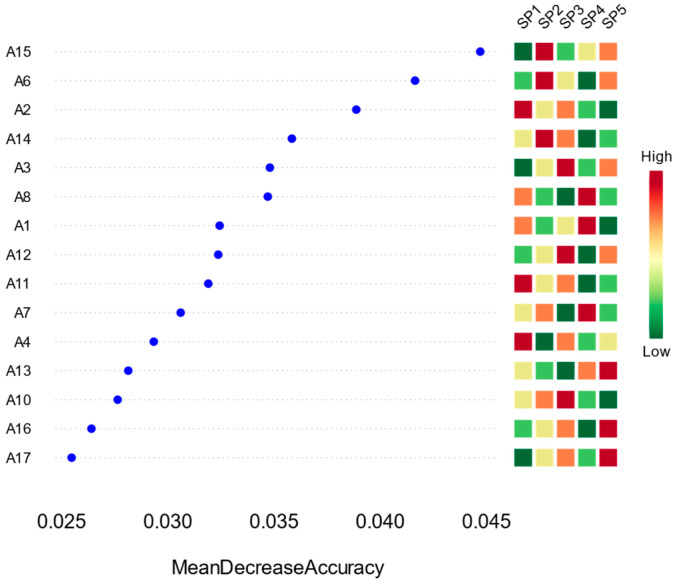
Discriminant importance and abundance distribution of phytoplankton dominant species (SP) based on the Random Forest Model (RFM); on the left side of the figure, the discriminant importance of dominant species (SP) in the RFM is ranked according to the declining value of the average accuracy, and on the right side of the figure is the standardized abundance heatmap of the dominant species in the 17 sampling points (A1–A17).

From the results in Figure 11, SP1 ranked the highest in importance. It contributed the most to enhancing the model’s classification ability, with a much higher decline in mean accuracy than the other dominant species, suggesting that it was the most indicative of community differentiation. SP2, SP3, and SP4 also had high importance scores and were the next most critical variables for the model to discriminate between differences in the sample sites. In contrast, SP4 and SP5 ranked the lowest, suggesting that they were more evenly distributed among different sample sites and were less responsive to environmental change, with limited significance.

The abundance heatmap shows that SP1 exhibits high abundance (in the red-orange color) in several sample points, such as A2, A4, and A11, suggesting that it has a strong adaptive ability to local environments. The abundance of SP2 and SP3 is also higher in some of the sample points, but with more pronounced spatial variations.

Overall, the random forest model successfully identified a group of dominant species representative of community differentiation. These species had large differences in abundance and strong indications among the sample sites, reflecting that they may be strongly regulated by environmental factors and have high sensitivity to ecological responses. On the other hand, the subordinate dominant species were evenly distributed and weakly responsive, and their roles in the ecosystem were relatively weak.

## 4. Discussion

### 4.1. Characterisation of Phytoplankton Community Structure and Analysis of Dominant Mechanisms

A total of 209 phytoplankton species belonging to seven phyla were identified in a reservoir in Ili, Xinjiang, showing a high species richness and structural complexity of the phytoplankton community in the reservoir in the arid zone. However, from the perspective of community composition, the water body showed obvious ‘oligarchic’ characteristics, i.e., a few taxa dominated the number of species, and the distribution of the community was highly concentrated, with Bacillariophyta, Chlorophyta, and Cyanobacteria as the main taxa, accounting for 92.34% of the total species number. The proportion was as high as 92.34%, of which the diatom phylum alone accounted for 40.67%, the dominant taxon. This result is consistent with other water bodies in arid or semi-arid zones, indicating that taxa with stronger environmental adaptability are more likely to form a dominant position under limited resources and environmental fluctuations.

With siliceous cell walls and strong environmental tolerance, diatoms are usually dominant in low-nutrient, high-transparency, or highly mobile water bodies [18]. This is supported by their high percentage in the present study reservoirs, which may be related to the fact that the water bodies were in a relatively clean, high-transparency state with moderate nutrient inputs. Green algae, although slightly inferior to diatoms, were widely distributed in some water bodies due to their wide adaptability and high metabolic activity, reflecting the relatively abundant supply of nitrogen and phosphorus resources or the tendency of local eutrophication in some areas of the reservoir [19,20]. The proportion of cyanobacteria was 18.18%, which was lower than that of diatoms and green algae. However, as a group highly sensitive to environmental conditions such as temperature and light, its distribution also suggested that the water body might have the conditions to promote the growth of cyanobacteria in some spatial and temporal scales [21].

Regarding density and biomass, the phytoplankton community is separated into ‘quantitative’ and ‘qualitative’ dominant groups. In terms of density, the diatom phylum was dominant, and its cell number was much higher than that of the other phyla, which showed that it was widely distributed in the whole water body and its population was prosperous. In terms of biomass, significant differences were observed, with the cyanobacteria phylum becoming the most dominant biomass contributor, which might be related to its larger cell size and sparse community structure [22,23]. In contrast, despite the abundance of diatoms, the overall biomass of diatoms did not form an absolute advantage due to their smaller cell structure and lower unit mass [24,25].

This separation of ‘quantitative advantage’ and ‘qualitative advantage’ is common in freshwater ecosystems and reflects the differences in ecological functions and resource utilization strategies of different groups. Diatoms rapidly occupied the water body resource space with a high reproduction rate and good planktonic ability [26]. They became the quantitative basis of primary productivity. In contrast, cyanobacteria showed competitive advantages in photosynthetic efficiency and buoyancy regulation. They became an important driving factor influencing the nutrient cycle of the water body and the feeding behavior of zooplankton [27]. The segregation characteristics further revealed the complex relationship between the structure and function of the phytoplankton community and provided directional guidance for the subsequent food web analysis.

The geographical distribution of the dominant species structure is also noteworthy. This study found that the distribution of dominant species in different sampling sites differed significantly, showing strong spatial heterogeneity. For example, the dominance of lake-born *Pseudourostyla crassipes* was high but mainly concentrated in a few eutrophic sites, indicating that it was more likely to form high biomass aggregation in areas with high nutrient input and weak water exchange capacity [28]. At the same time, it was less likely to occur in sites with frequent water exchange or poor nutrient conditions. This ecological niche adaptation not only determines the spatial distribution pattern of dominant species but also indirectly influences zooplankton’s distribution and feeding choice, which then feeds back on the material cycle and energy flow in the water column [29].

To better contextualize our findings, the abundance and biomass patterns of the dominant phytoplankton phyla were compared with those reported in similar lentic ecosystems and historical studies from the Ili River Basin. The dominance of Bacillariophyta and Chlorophyta in terms of density has also been reported in other temperate reservoirs, such as in the middle reaches of the Yellow River and the Danjiangkou Reservoir in central China, where diatoms and green algae prevail under moderate nutrient and light conditions [30,31]. However, the exceptionally high biomass of Cryptophyta observed in this study differs from most reports and may be attributed to regional hydrodynamic conditions and local nutrient pulses. In contrast, previous studies in the Ili region primarily reported dominance by Cyanobacteria during summer, whereas our results show a relatively low cyanobacterial density and biomass, possibly reflecting recent improvements in nutrient management and reduced thermal stratification.

In addition, the community structure of ‘dominated by a few taxa and sparsely populated by most taxa’ presented in this study is of great ecological significance. In reservoir ecosystems with strong resource constraints and environmental stresses, the dominant taxa usually have strong resource utilization ability, environmental adaptability, and competitive advantages. In contrast, the non-dominant taxa, although numerous, are scattered and have limited ecological contributions. This structure is conducive to the stability of the ecosystem to maintain its basic functions, but also suggests that the diversity redundancy of the system is relatively low, and there is a structural vulnerability in the face of disturbances [32].

### 4.2. Community Response Mechanisms and Ecological Niche Differentiation Driven by Environmental Factors

Temporally, environmental factors in August and October showed distinct seasonal differences in correlation patterns. Water temperature (WT) and oxidation-reduction potential (ORP) were negatively correlated in both months, with a stronger correlation observed in October, suggesting that the cooling climate enhanced the redox regulation in the water body. Similarly, the correlation between ORP and pH was strongly negative in August but only moderately negative in October, indicating a diminished influence of redox status on pH in autumn. These patterns may be attributed to the seasonal decline in self-purification capacity and microbial activity, highlighting the indirect regulatory role of temperature on water chemistry.

The correlation between dissolved oxygen (DO) and WT also differed significantly between the two periods. In August, the DO-WT correlation was strongly negative (r = −0.82), suggesting that high temperatures substantially suppressed oxygen solubility, possibly due to reduced photosynthesis or increased microbial respiration. In contrast, the correlation in October was weak (r = 0.16), indicating that DO variation in cooler seasons was more likely influenced by factors such as nutrient availability and hydrological dynamics.

Additionally, salinity (Sal), electrical conductivity (EC), and total dissolved solids (TDS) remained highly positively correlated across both seasons, reflecting the seasonal stability of inorganic ion concentrations, likely governed by external inputs or reservoir recharge regimes. The negative correlations between DO and EC/TDS were more pronounced in August, suggesting that under high-temperature conditions, elevated mineral content may further constrain oxygen availability—posing a significant environmental stressor to phytoplankton in arid-region water bodies [33].

Spatially, heat maps of MOTU (Morphological Operational Taxonomic Unit) and SP (dominant species) abundance across 17 sampling sites highlighted structural differences between taxonomic levels. MOTU1 was the most dominant across most sites, exhibiting broad spatial prevalence and ecological adaptability. MOTU2 and MOTU5 showed moderate abundance in a few localized sites. However, they were less widespread than MOTU1, whereas MOTU4, MOTU6, and MOTU7 were consistently low in abundance, indicating limited adaptive capacity or narrow ecological niches under current water conditions.

Compared to MOTUs, the spatial distribution of SPs demonstrated stronger localization. Most SPs had low abundance, with only SP1 and SP4 showing relative dominance at a few sites. This suggests that dominant species respond more strongly to localized environmental factors such as nutrient inputs, flow velocity, and water depth [34]. This “MOTU-core, SP-local” pattern suggests that phytoplankton exhibit different ecological strategies at different taxonomic resolutions: core MOTUs are broadly adaptable and stable [35]. At the same time, dominant species are more constrained by microhabitat conditions.

Cluster analysis supported this hierarchical difference, as MOTU and SP groups formed separate clusters, indicating distinct environmental response pathways. MOTUs dominated across multiple sites, showing system-wide influence. At the same time, SPs exhibited site-specific peaks, reinforcing the concept of functional complementarity between core and peripheral species in maintaining ecosystem stability under complex environmental scenarios.

### 4.3. Phytoplankton Response Patterns at Different Ecological Levels

In the PCA space, MOTUs exhibited a wider distribution, with sampling points stretching notably along the principal axis, indicating greater ecological plasticity and structural heterogeneity at the community level in response to environmental gradients. This variability likely stems from intrinsic differences among taxonomic groups in life history strategies, photosynthetic efficiency, nutrient requirements, and sensitivity to hydrodynamic conditions [36]. For instance, diatoms may prefer clear, slow-flowing, and nutrient-poor waters. At the same time, green algae and cyanobacteria are often more responsive to temperature and eutrophication, thus showing different spatial trajectories in the PCA plot.

In contrast, the distribution of SPs was more concentrated, indicating that a few dominant species with high abundance in the samples responded more uniformly to environmental gradients. This consistency suggests that dominant species exhibit stronger convergence in response to specific environmental variables—such as salinity, pH, and total dissolved solids (TDS)—and have formed localized ecological adaptations, securing stable dominance [37,38]. These findings are consistent with earlier heatmap and clustering analyses, which indicated that while the overall community structure demonstrates broad adaptability, dominant species exhibit more localized and selective responses.

Redundancy analysis (RDA) provided further insight into the relationships between community structure and environmental variables. MOTU1 was closely aligned with ORP, suggesting that it is highly sensitive to the redox conditions of the water body and may prefer oxygen-rich or highly oxidizing environments, making ORP its primary ecological driver. MOTU2 and MOTU3 were located in transitional zones between multiple environmental vectors, indicating that their response patterns are jointly influenced by several factors. This shows ecological neutrality or a broader adaptive strategy. Other MOTU groups clustered near the center of the ordination plot, with shorter arrow vectors, suggesting a weaker response to environmental gradients or a more uniform distribution across water bodies, possibly due to broader tolerance ranges. Among environmental variables, ORP, pH, and WT had the longest and most stable arrow vectors, indicating that they were the most influential drivers of community structure. ORP consistently aligned with multiple MOTU distributions. As an integrated indicator of redox conditions, ORP influences algal metabolism, photosynthetic efficiency, and microbial activity, thereby playing a central role in shaping community succession [39,40].

The seasonal environmental patterns observed in the Ili Reservoir were closely linked to regional climatic conditions. In August, higher temperatures and increased solar radiation, combined with relatively low water levels, created favorable conditions for fast-growing taxa like Chlorophyta. In contrast, the cooler and more turbid conditions in October, resulting from seasonal precipitation and surface runoff, were associated with a shift toward Bacillariophyta dominance. These seasonal transitions are consistent with broader climatic patterns in arid northwest China, where summer evaporation and autumn cooling exert strong effects on aquatic primary productivity and community dynamics.

Additionally, Sal, EC, TDS, and DO clustered in a similar directional quadrant of the RDA plot, primarily influencing the sampling points in the lower-left region, suggesting that these sites are characterized by higher mineralization, ionic concentration, or possible eutrophic conditions. This pattern reflects substantial environmental differentiation at these sites, potentially influenced by agricultural runoff, local geological features, or anthropogenic disturbance. The significant deviation of site A12, in particular, points to a distinct ecological niche or marginal ecosystem, warranting special attention in future monitoring and management.

### 4.4. Machine-Learning-Based Detection of Core Taxa and Their Environmental Implications

According to the variable importance rankings from the model output, MOTU1 exhibited the highest contribution among all taxa, with its mean decrease in accuracy substantially exceeding those of other MOTUs. This suggests strong indicative and representative value in differentiating community structures across sites. MOTU2 through to MOTU5 also showed high importance scores and were identified as key discriminant variables following MOTU1. These taxa had relatively high abundances and demonstrated greater sensitivity to ecological variation, implying their potential roles in community structuring, primary productivity, and competitive resource utilization.

In contrast, MOTU4 and MOTU5 showed importance scores close to zero, indicating minimal contribution to model accuracy. Heatmap analysis revealed that these taxa maintained consistently low abundances across sites, with minimal spatial differentiation. These low-abundance, low-importance taxa may be considered “ecological edge species” or “rare taxa,” which, while contributing to community diversity, play limited roles in functional expression or structural assembly under current environmental conditions [41]. The spatial distribution of MOTUs also showed pronounced heterogeneity. MOTU1 displayed high abundance across multiple sites, particularly in areas with stable environmental conditions or sufficient resource availability, but was relatively sparse at sites such as A1 and A3, suggesting specific environmental preferences or limitations. Similarly, MOTU2 and MOTU3 exhibited localized dominance in certain sites but had less widespread distributions than MOTU1. This “core taxa plus local dominance” pattern reflects strong spatial responsiveness at the taxonomic level and provides insight into mechanisms underlying community dynamic stability [42].

At the dominant species level (SPs), the random forest model also identified a subset of species with high discriminative power. SP1 ranked highest in importance, serving as the most influential indicator for distinguishing community differences among sampling sites. SP2, SP3, and SP4 scored relatively high as secondary key variables. Unlike MOTUs, SPs exhibited more localized spatial distributions, characterized by high abundances at specific sites and low abundances elsewhere. This pattern indicates that dominant species generally possess strong site-specific adaptability, narrow ecological niches, and high sensitivity to environmental drivers, enabling them to attain dominance under specific habitat conditions [43].

In contrast, low-ranking SPs (e.g., SP12–SP15) had uniformly low abundance across all sites, with little spatial variation, reflecting weak responsiveness to environmental changes. While these species were present throughout, their low abundance and stable distribution made them less effective in differentiating community structure. This suggests that SP-level indicators may be more suitable for identifying localized ecological conditions rather than characterizing broad-scale community patterns [44].

A comparative analysis of MOTUs and SPs in the random forest model reveals a clear functional complementarity. MOTUs represent broader taxonomic structures with higher ecological generality and environmental tolerance, which are suitable for identifying foundational ecological patterns and ecosystem-level responses [45]. In contrast, SPs capture fine-scale variability and serve as sensitive indicators of localized environmental anomalies or unique ecological states. The integration of both levels provides multi-tiered evidence for understanding the roles of phytoplankton in functional maintenance and response to environmental stressors [46].

Furthermore, the observed correspondence between “high-importance taxa” and spatial heterogeneity underscores their relevance for future ecological monitoring and water quality assessment. Tracking changes in the abundance and distribution of key taxa, such as MOTU1 and SP1, can be an early-warning system for detecting eutrophication, water quality degradation, or ecosystem imbalance [47]. Meanwhile, low-importance but consistently present taxa may serve as baseline reference indicators for evaluating system stability.

## 5. Conclusions

A total of 209 phytoplankton species were identified in the study area, belonging to seven taxonomic phyla and exhibiting a distinct “oligarchic” community structure. Diatoms, green algae, and cyanobacteria dominated the community composition, collectively accounting for over 92% of the total species. Diatoms were predominant regarding cell density, while cyanobacteria contributed most to biomass, reflecting a clear decoupling between “numerical dominance” and “biomass dominance.” Analysis of dominant species further identified *Dolichospermum flosaquae* as having the highest dominance index, suggesting strong environmental adaptability and ecological indicator potential.

From the perspective of environmental response, the correlation structure of water physicochemical parameters in August and October showed notable seasonal differences, highlighting the significant regulatory effect of temporal variation on aquatic ecological processes. Phytoplankton at the taxonomic group level (MOTUs) showed strong negative correlations with oxidation-reduction potential (ORP), dissolved oxygen (DO), electrical conductivity (EC), and total dissolved solids (TDS), indicating their sensitivity to mineralization and redox conditions. In contrast, dominant species (SPs) were more positively correlated with salinity, pH, and TDS, suggesting selective responses to environmental gradients at different ecological levels.

Principal component analysis (PCA) and redundancy analysis (RDA) revealed distinct MOTUs and SPs distribution patterns along environmental gradients. SPs exhibited higher aggregation along specific axes, whereas MOTUs showed broader ecological niche responses. Further analysis using heatmaps and hierarchical clustering demonstrated that a few core MOTUs maintained stable dominance across multiple sites. At the same time, SPs showed more localized distributions, supporting the spatial structure pattern of “widely distributed core taxa and locally dominant species.”

The random forest model further identified key taxa and dominant species with strong discriminatory power in differentiating spatial patterns. MOTU1 and SP1 exhibited the highest importance scores, indicating their central ecological roles and potential as priority targets for future environmental monitoring and indicator species selection.

## Figures and Tables

**Table 1:** Dominant phytoplankton species in reservoirs of the Ili Region, Xinjiang.

Phyla	Species	Dominance	Codes
Bacillariophyta	*Nitzschia palea* (Kützing) W. Smith	0.07	SP1
Bacillariophyta	*Achnanthes exigua* Grunow	0.02	SP2
Bacillariophyta	*Synedra acus* Kützing	0.02	SP3
Bacillariophyta	*Cymbella cistula* (Ehrenberg) Kirchner	0.02	SP4
Chlorophyta	*Limnothrix redekei* (Van Goor) Meffert	0.24	SP5
Bacillariophyta	-	-	MOTU1
Chlorophyta	-	-	MOTU2
Cyanobacteria	-	-	MOTU3
Chrysophyta	-	-	MOTU4
Cryptophyta	-	-	MOTU5
Euglenophyceae	-	-	MOTU6
Dinophyta	-	-	MOTU7

**Table 2:** Comparison of key water quality parameters between August and October.

Parameter	Unit	August (Mean ± SD)	Range (Aug)	October (Mean ± SD)	Range (Oct)	ANOVA F-Value	*p*-Value
WT	°C	21.82 ± 2.40	18.11–26.20	14.62 ± 1.90	11.20–17.80	115.72	<0.001
pH	–	7.93 ± 0.25	7.52–8.45	7.61 ± 0.18	7.29–7.95	14.83	<0.01
EC	μS/cm	650 ± 81	505–812	683 ± 75	545–810	2.57	0.118
Sal	ppt	0.34 ± 0.04	0.27–0.42	0.36 ± 0.03	0.30–0.42	2.02	0.163
TDS	mg/L	437 ± 61	331–538	452 ± 59	355–547	1.78	0.191
ORP	mV	124 ± 27	85–179	141 ± 31	96–188	5.94	0.024
DO	mg/L	6.42 ± 1.14	4.41–8.26	7.86 ± 0.92	6.35–9.33	28.73	<0.001

## Data Availability

The data supporting this study’s findings are available from the corresponding authors upon reasonable request.
